# Social protection: potential for improving HIV outcomes among adolescents

**DOI:** 10.7448/IAS.18.7.20260

**Published:** 2015-12-02

**Authors:** Lucie D Cluver, Rebecca J Hodes, Lorraine Sherr, F Mark Orkin, Franziska Meinck, Patricia Lim Ah Ken, Natalia E Winder-Rossi, Jason Wolfe, Marissa Vicari

**Affiliations:** 1 Centre for Evidence-Based Intervention, Department of Social Policy & Intervention, University of Oxford, Oxford, UK; 2 Department of Psychiatry and Mental Health, University of Cape Town, Cape Town, South Africa; 3 AIDS and Society Research Unit, Centre for Social Science Research, University of Cape Town, Cape Town, South Africa; 4 Department of Historical Studies, University of Cape Town, Cape Town, South Africa; 5 Health Psychology Unit, Department of Infection & Population Health, University College London, London, UK; 6 School of Clinical Medicine and DST-NRF Centre of Excellence in Human Development, University of the Witwatersrand, Johannesburg, South Africa; 7 HIV and AIDS Section, UNICEF, New York, USA; 8 UNICEF Regional Office for Eastern and Southern Africa, Nairobi, Kenya; 9 Office of HIV/AIDS, Bureau for Global Health, US Agency for International Development, Washington, DC, USA; 10 Collaborative Initiative for Paediatric HIV Education and Research (CIPHER), International AIDS Society, Geneva, Switzerland

**Keywords:** social protection, HIV/AIDS, adolescents, HIV prevention, adherence

## Abstract

**Introduction:**

Advances in biomedical technologies provide potential for adolescent HIV prevention and HIV-positive survival. The UNAIDS 90–90–90 treatment targets provide a new roadmap for ending the HIV epidemic, principally through antiretroviral treatment, HIV testing and viral suppression among people with HIV. However, while imperative, HIV treatment and testing will not be sufficient to address the epidemic among adolescents in Southern and Eastern Africa. In particular, use of condoms and adherence to antiretroviral therapy (ART) remain haphazard, with evidence that social and structural deprivation is negatively impacting adolescents’ capacity to protect themselves and others. This paper examines the evidence for and potential of interventions addressing these structural deprivations.

**Discussion:**

New evidence is emerging around social protection interventions, including cash transfers, parenting support and educational support (“cash, care and classroom”). These interventions have the potential to reduce the social and economic drivers of HIV risk, improve utilization of prevention technologies and improve adherence to ART for adolescent populations in the hyper-endemic settings of Southern and Eastern Africa. Studies show that the integration of social and economic interventions has high acceptability and reach and that it holds powerful potential for improved HIV, health and development outcomes.

**Conclusions:**

Social protection is a largely untapped means of reducing HIV-risk behaviours and increasing uptake of and adherence to biomedical prevention and treatment technologies. There is now sufficient evidence to include social protection programming as a key strategy not only to mitigate the negative impacts of the HIV epidemic among families, but also to contribute to HIV prevention among adolescents and potentially to remove social and economic barriers to accessing treatment. We urge a further research and programming agenda: to actively combine programmes that increase availability of biomedical solutions with social protection policies that can boost their utilization.

## Introduction

Adolescent HIV support has misfired throughout the epidemic, caught in a wrinkle of under-provision and misunderstanding, with avoidable HIV infection and death as the result. Antiretroviral therapy (ART) and condoms provide the means for adolescents to live with HIV and to prevent transmission. However even where these technologies are available, high rates of HIV infection, morbidity and mortality persist, particularly in Southern and Eastern Africa. HIV infections among adolescents in the region remain at 440 per day [1] with approximately 2.1 million adolescents (aged 10 to 19) living with HIV, the majority in Southern Africa [2]. Worldwide, adolescents are the only age group in which AIDS-related deaths are not decreasing [1].

Why is this? Evidence shows that technologies, despite their efficacy, are not being accessed or used consistently. Biomedical responses are imperative but not sufficient, and they remain obstructed by weak procurement and supply management systems, human resources shortages and the high cost of particular treatment regimens and diagnostics [3, p. 10]. The UNAIDS 90–90–90 commitment highlights the importance of achieving better coverage of HIV testing, ART initiation and retention in effective care. However, its focus is principally on the provision of medical technologies within the health sector, rather than on the expansion of other services and interventions from within the overlapping spheres of social development and education [3]. Similar efforts and commitments related to increasing access to and uptake of prevention services among adolescents are needed if the goals of HIV prevention and treatment programs are to be achieved [3].

Reported rates of girls’ condom use at last high-risk sex was less than one-third in nine countries in Southern and Eastern Africa [4] and even lower in poor households and rural areas [5]. ART adherence also remains a major challenge: in a nine-country regional study, adolescent adherence was between 7 and 20% [6,7]. A recent trial of pre-exposure prophylaxis (PrEP) in Zimbabwe, South Africa and Uganda was halted early due to adherence rates of 25 to 30%, which were even lower among younger, single women [8].

Consistent condom and PrEP use and ART adherence are global challenges. For adolescents in resource-limited settings, individual barriers are exacerbated by socio-economic, environmental and structural constraints. Adolescence is a period of emotional-social development, growing independence and changing relationships with families, peers and romantic partners [9]. Associations between HIV infection, poverty and inequality are complex [10–12], but a growing body of research has established that social and structural deprivation, often with gendered aspects, are key drivers of adolescent HIV infection and mortality [13]. These deprivations include poverty and exclusion [14], income shocks [15,16], mental health distress, stigma [17], harsh parenting and abuse [18,19]. Exposure to multiple stressors can have cumulative effects, maximizing HIV-related risks [19]. In addition, HIV risk behaviours are primarily extra-clinical, occurring in the social spaces beyond the health system where adolescents live, have fun and take risks. HIV prevention programmes that focus on individual behaviours but do not account for their socio-ecological basis are likely to have limited efficacy [20]. This paper examines a set of interventions that aim to address socio-economic vulnerabilities among young people, assessing the quality and extent of the evidence base for social protection in contributing to our goals of reaching the 90–90–90 targets.

## Discussion

Interventions that function beyond the clinic, incorporating broader populations than patients alone, have begun to capture the attention of policymakers [21,22]. There is increasing interest in social protection as a potential intervention to improve HIV prevention and treatment outcomes in adolescence, by ameliorating the socio-economic deprivations that increase risks. Moreover, interventions that combine clinical and social care may have wider benefits for populations beyond HIV effects [23].

### Social protection and adolescent HIV prevention

*Social protection* is often understood as the transfer of cash to poor and vulnerable populations [24], but it is conceptually more expansive. UNICEF's definition for social protection incorporates “the set of public and private policies and programmes aimed at preventing, reducing and eliminating economic and social vulnerabilities to poverty and deprivation.” [25]. This can include a range of provisions. For example Devereux operationalizes social protection as emergency food aid, public works projects and agricultural subsidies [26]; de Haan highlights non-contributory schemes targeting chronic poverty [27]; and Haushofer notes increasing interest in health insurance and savings support [28]. Social protection programmes can include economic, social and psychosocial provisions administered by governments, NGOs or communities, or combinations of these modalities.

A new body of evidence shows that social protection can reduce risks of HIV infection in sub-Saharan Africa. While extensive evidence from Latin America and Africa links social protection to wider facets of wellbeing [28], research with HIV outcomes has largely focused on specific social protections of cash transfers, food provision and psychosocial care. In a randomized trial in Malawi, both unconditional and educational-conditioned cash transfers reduced HIV prevalence among girls [29]. A cluster randomized trial in Kenya and two propensity-matched studies in South Africa (one on a national cross-sectional dataset and another on a two-province longitudinal study, *n*=3500) all demonstrated associations between national unconditional cash transfer programmes and reduced HIV-infection risks among adolescents, particularly girls. These include reductions in sexual debut, pregnancy, age-disparate sex and transactional sex [30–32].

In the next year, further findings will emerge from the Transfer Project's [33] cluster randomized trials of national social cash transfers in Malawi, Zambia and Zimbabwe. Critiques of this research note that some findings are limited by a lack of HIV-incidence biomarkers, instead using proxies of self-reported behaviour, pregnancy and HIV prevalence. The evidence from South Africa uses propensity score matching rather than randomized trials (due to existing national cash transfer programmes) and there are ongoing debates concerning whether we should prioritize the real-world applicability of testing impacts of existing programmes or the causal reliability of randomized trials.

**Figure 1:**
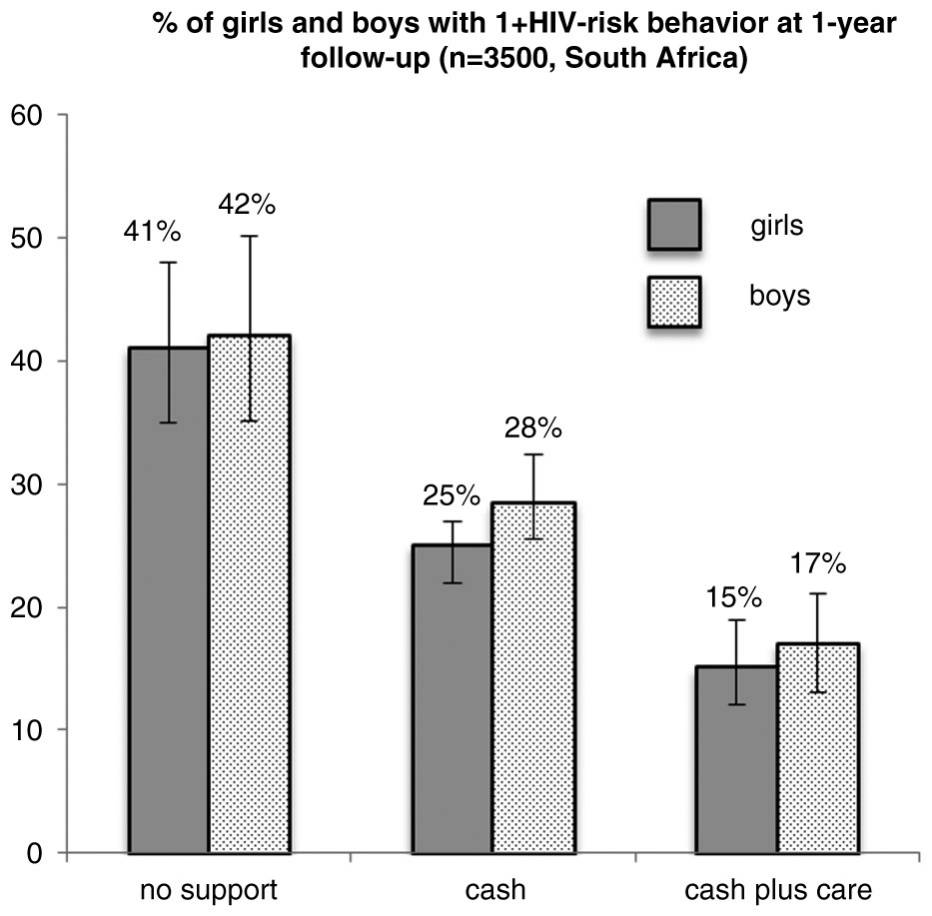
Impacts of cash and care provision on HIV-risk behaviour among adolescents in South Africa (marginal effects models, controlling for covariates) [34].

Research shows that cash transfers are not the only form of social protection with HIV-prevention effects. In South Africa, the combination of social welfare grants or school feeding with positive parenting or teacher support (“cash plus care”) was shown as more effective than cash transfers alone in reducing adolescent risk behaviour (see Figure 1) [34].

Indeed, new data demonstrate that *combination social protection* (providing specific combinations of cash transfers, school feeding, free schools, parental supervision and teacher support) shows cumulative risk-reduction effects among adolescents [35]. Such combinations appear to be most effective among the most difficult to reach groups. In Uganda, a combination programme of economic support (matched savings), educational support and mentoring improved mental health and educational attainment and reduced sexual risk-taking intentions among younger children [36,37]. Indeed, the strong prevention effects of keeping adolescents in school and of providing free schooling, school feeding and supportive teachers [38] suggest that social protection for adolescents should be expanded beyond cash plus care to include “cash, care and classroom.”

### Social protection and adolescent treatment

HIV-positive adolescents include two co-existing groups with shared and unique risks [39]. Survivors of vertical infection are growing up with lifelong exposure to HIV, high risk of cognitive impairment [40] and parenting environments often characterized by illness and death. Adolescents who acquire HIV through sexual transmission may differ in behavioural profiles, but both groups are strongly influenced by family-level and structural factors [41]. Recent systematic reviews [7,42] identify risk factors for ART non-adherence among adolescents, including social and structural deprivations of poverty, disorganized families, caregiver–child conflict and mental health problems [43]. A recent situation analysis in 23 sub-Saharan African countries found that socio-economic challenges including transportation costs and food insecurity were the greatest barriers to adolescent treatment and care [44]. Research has identified protective impacts of early disclosure to adolescents of their HIV status [45], but overall there is very limited research on how to improve adolescent adherence.

Evidence from the region suggests that social protection has the potential to be an effective intervention for HIV-positive adolescents and thus could contribute directly to achieving the UNAIDS 90–90–90 commitments. In South Africa, adolescent ART adherence was improved by social protection interventions of school feeding programmes, parental monitoring and social support [2]. A study of cash transfers for clinic transportation among adults in Uganda showed improved adherence and retention in care [46]. Several studies of mentorship mother programmes have shown improved PMTCT adherence [47–50], suggesting both a potential impact for adolescent mothers and prevention of child infection. In Tanzania, an ongoing study among adults is testing whether combinations of nutrition counselling, food assistance and unconditional cash transfers can promote ART adherence [51]. In Uganda an adolescent-focused randomized trial is currently testing effects on adherence of family savings with evidence-based family-support programming [52]. The recent WHO consultation on the upcoming 2015 treatment guideline update identified social protection (including food vouchers) as a key priority area for adolescents [53].

### Clearing confusion: cash incentives, conditional cash transfers and social protection

Cash incentive programmes are often conflated with social protection, but some differ fundamentally in their conceptual approach. Unconditional social protection premises that sexual decisions in low-resource contexts such as sub-Saharan Africa are constrained by structural deprivations such as poverty and violence, which need to be mitigated to allow healthy decision-making. In contrast, cash incentives premise that adolescent HIV risks are driven primarily by behavioural choice and can therefore be changed by provision of alternative (cash) rewards [54]. Pettifor distinguishes cash incentive programmes that are dependent on specific sexual behavioural adaptations as “downstream programmes” [55], tethered to a more individualist orientation for health promotion.

The evidence from trials of cash incentives is mixed. No studies have examined cash incentives among adolescents under the age of 18. No known studies have examined cash incentives for ART adherence in adolescents. In Tanzania among adults aged 18 to 30, a high cash amount (but not a lower amount), which was conditional on negative results of quarterly testing for sexually transmitted infections, reduced sexually transmitted infection (STI) prevalence compared to no-cash controls [54]. In Lesotho, a lottery-based system for 18 to 32 year olds, also based on negative results of STI testing, showed reductions in HIV incidence, but there remain questions about introducing gambling as a behavioural mechanism. In the United States, studies with adult drug-using HIV-positive populations have shown improvements [56] but a recent RCT showed no effects [57]. All evidence to date on cash incentives is from researcher-run programmes in randomized trials. Such trials provide greater confidence in causal relationships but may represent proof of concept rather than scalability and require further effectiveness testing as well as testing with adolescent populations.

A major concern with incentives is the complexity and costs associated with implementation, policing conditions and applying sanctions, which have been questioned as potentially unfeasible in resource-constrained countries with limited administrative capacity. In addition, critics raise ethical concerns about removing sources of income from poor adolescents in periods of acute vulnerability, such as HIV diagnosis. There are further questions about the validity of assumptions that STI infection represents a behavioural choice, when evidence shows high rates of rape and coerced sex among adolescent girls [58,59].

Somewhere between the two approaches of social protection and cash incentives is a third option of conditional cash transfers, focused on incentivizing behaviour to utilize services that will reduce structural drivers of HIV risk [55]. This approach is based on an understanding that the cash will serve to ensure the adoption of well-established beneficial behaviours such as school attendance [38]. Such cash transfers have been used for non-HIV behaviour change in national programmes in Latin America. These are based on robust evidence that schooling is protective against HIV infection, particularly for girls [60]. Among adults in Kenya, incentives for voluntary counselling and testing showed increased uptake [61]. New findings from two randomized trials in South Africa were reported in July 2015, both with promising findings. An individually randomized trial of cash transfers conditional on girls’ school attendance (ages 13 to 20) found reduced self-reported HIV-risk behaviours [62]. A cluster randomized trial of cash transfers for adolescents in early high school (grades 9 to 10, ages around 15 to 17), conditional on a range of behaviours including attendance at extracurricular activities, school exam success and HIV testing, showed reductions in HSV-2 incidence [63]. However, both trials had lower than expected overall HIV-incidence rates in the adolescent age group and may have had power thus reduced for this biomarker, reflecting challenges of testing HIV incidence as a primary outcome for adolescent programmes.

It is important to consider supply issues if conditionalities were to be taken to scale. In Tanzania, schooling conditions for secondary-age adolescents had to be removed due to insufficient schools to absorb the increased demand. These programmes show potential, but we also need to assess outcomes where conditionality may remove support from those at greatest risk for HIV transmission as well as for non-compliance to conditions. Further research is required to determine whether conditionality improves outcomes in comparison to unconditional cash transfers. Only one study date compares them: in Malawi, cash transfers reduced HIV prevalence and HSV prevalence among girls, but showed no differences between unconditional and conditional transfers [29].

### Is social protection affordable, acceptable and scalable?

The introduction of social grant systems has risen dramatically in Southern and Eastern Africa. Today, most countries in the region have implemented social protection programmes, although many remain small-scale. South Africa leads this trend, with over 16,640,000 grants disbursed monthly as of March 2015 – an economic lifeline for many [64]. The new DREAMS partnership, an initiative of PEPFAR, the Gates Foundation and the Nike Foundation, aims to reduce HIV infections among adolescent girls and young women in the region. Based on evidence synthesis, DREAMS includes a “combination prevention” package of biomedical and behavioural interventions, such as condom provision, HIV testing and PrEP in selected contexts, together with social protection interventions such as parenting programmes, school-based violence prevention, cash transfers and educational subsidies [65]. UNAIDS has identified social protection as a critical enabler in HIV prevention and is developing tools to assess country-level social protection programming and its potential for HIV synergies. There is remarkable potential for research, sustainability and expansion of social protection programmes.

However is social protection scalable and sustainable in the region? Seekings identifies that barriers to national social protection programmes are often neither administrative nor fiscal: both the ILO and World Bank show that most African countries can afford to expand their social protection floors [66]. Instead, barriers are often social and political attitudes, based on mutable perceptions of who is deserving of support [67]. The views of political elites, electorates, advocacy groups and international agencies may all have influence [66], although studies show high levels of acceptability at a population level [68]. Thus, programmes must intersect with the needs of a society in ways that enhance their acceptability and efficacy. It is also argued that social protection must be seen by governments of emerging economies as a developmentalist tool [27] and that it should be based on the availability and sustainability of local services [69] in order to be acceptable and feasible. Studies in Southern and Eastern Africa have found cost-effectiveness of national cash transfers and school support for adolescents in generalized epidemics [70], with long-term savings on avoidance of future negative outcomes. As the sustainable development goals move away from an HIV focus towards broader aims and integrated responses, there may be increasing rationale for programming that demonstrates evidence of multiple health and development outcomes [71]. The STRIVE consortium demonstrated that co-financing from multiple government departments that benefit from social transfers can result in manageable budgetary commitments [72,73], and this is reflected in trends towards domestically funded social protection programmes in the region.

### The next research agenda: combination prevention and protection

There are pockets of vulnerable populations across the globe, but this must not obscure the reality that adolescents in Southern Africa and in particular girls and young women bear the heaviest burden of HIV risk.

However, research and interventions among these populations have been conducted primarily in the global north. Delany-Moretlwe's review of health services for young key populations points to the lack of research on barriers and facilitators of comprehensive health care among adolescents in Africa, with less than 10% of studies cited located in an African setting. Lall *et al*. highlight the urgent need for research on ART adherence among HIV-positive adolescents and youth, with most studies and programming on ART adherence and retention in care among adolescents conducted in Northern America [74,75]. It will be important to understand how social protection policies interact with availability and acceptability of sexual and reproductive health services in high-epidemic contexts and how that may affect adolescent girls and young women in particular. We must also be cautious of assuming transferability of findings across contexts, both between and across high and low resource settings. Research must grapple with the situation-dependent and context-specific nature of behaviour and identity and how these are negotiated, produced, gendered and constructed.

It is increasingly clear that public health interventions must be adapted to local contexts if they are to succeed on a sustained basis [76]. Evidence suggests that social protection may be an effective facilitator of biomedical HIV prevention and treatment programming. Research to date suggests good evidence on cash transfers, emerging evidence on other forms of social protection and demonstrable increased effects of combining specific forms of social protection on reducing HIV risks for adolescents.

However no known research to date has examined effects of actively joining biomedical and social protection programming, “combination prevention and protection.” This presents an urgent research agenda, as highlighted by Celum and colleagues in a recent JIAS commentary [77]. HIV programming must be acceptable and effective for adolescents, both male and female [78]. It must also take into account the particularities of HIV risk and resilience in the unique contexts of Southern and Eastern Africa. Without access to condoms, circumcision, ART and potentially PrEP, there is little opportunity for adolescents to protect themselves and others. Social protection holds the potential to allow adolescents to use those opportunities.

## Conclusions

Despite the momentous progress that has been made in HIV treatment and prevention and the ambitious commitments to serving the needs of most at-risk populations, programmes remain inadequate to the major challenges adolescents face. While the global north has begun to speak of the “end of HIV,” those in the global south continue to grapple with endemic weaknesses in health systems, high incidence and the challenges of providing equitable, sustained access to HIV treatment and prevention. However even where there is access, severe structural and social deprivations experienced by the most vulnerable adolescents create barriers to uptake of and adherence to HIV prevention and treatment programmes. The UNAIDS publication on its 90–90–90 goals describes how the HIV epidemic galvanized global efforts to achieve greater equity, but emphasizes that, in order for these goals to be realized, the powerful momentum to provide programmatic responses must be sustained. Moreover, it highlights intolerable gaps in HIV treatment and service provision for adolescents in sub-Saharan Africa and the persistence of social and systemic challenges [3, pp. 7, 13].

Social protection that combines cash, care and classroom and other provisions can potentially reduce adolescent HIV-risk behaviour in the Southern and Eastern African region. Early evidence suggests that social protection may also increase adherence to PMTCT and ART for adolescents and youth. The next step in research and programming is to actively combine biomedical with social protection programmes (and potentially with efficacious behavioural programmes). With these combinations, access to biomedical technologies, social support and sufficient economic stability, we may be able to stem the adolescent epidemic.
